# Cancer chemoprevention with PV-1, a novel *Prunella vulgaris*-containing herbal mixture that remodels the tumor immune microenvironment in mice

**DOI:** 10.3389/fimmu.2023.1196434

**Published:** 2023-11-24

**Authors:** Qi Zhang, Xu Chen, Katie Palen, Bryon Johnson, Dinh Bui, Donghai Xiong, Jing Pan, Ming Hu, Yian Wang, Ming You

**Affiliations:** ^1^ Center for Cancer Prevention, Dr. Mary and Ron Neal Cancer Center, Houston Methodist Research Institute, Houston, TX, United States; ^2^ Department of Pharmacology & Toxicology, Medical College of Wisconsin, Milwaukee, WI, United States; ^3^ Department of Medicine, Medical College of Wisconsin, Milwaukee, WI, United States; ^4^ College of Pharmacy, University of Houston, Houston, TX, United States

**Keywords:** PV-1, chemoprevention, lung cancer, oral cancer, melanoma, tumor immune microenvironment

## Abstract

The herb *Prunella vulgaris* has shown significant immune-stimulatory and anti-inflammatory effects in mouse models. Here, the effects of a novel *Prunella vulgaris*-containing herbal mixture, PV-1, were examined in several mouse models for cancer, including chemically induced models of lung and oral cancers as well as syngraft models for lung cancer and melanoma. PV-1, consisting of extracts from *Prunella vulgaris*, *Polygonum bistorta*, *Sonchus brachyotus* and *Dictamnus dasycarpus*, exhibited no toxicity in a dose escalation study in A/J mice. PV-1 significantly inhibited mouse lung tumor development induced by the lung carcinogens vinyl carbamate and benzo[a]pyrene. PV-1 also hindered the induction of oral squamous cell carcinomas in C57BL/6 mice caused by 4-nitroquinoline-1-oxide. Flow cytometry analysis showed that PV-1 increased the numbers of CD8+ tumor-infiltrating lymphocytes (TILs) and increased the production of granzyme B, TNF-α, and IFN-γ by CD8+ TILs. PV-1 also suppressed granulocytic myeloid-derived suppressor cell numbers (g-MDSCs) and improved the anti‐cancer activity of anti-PD‐1 immunotherapy. These results indicate that PV-1 remodels the tumor immune microenvironment by selectively inhibiting g‐MDSCs and increasing CD8+ TILs within tumors, resulting in decreased immune suppression and enhanced cancer chemopreventive efficacy.

## Introduction

*Prunella vulgaris* (PV) is a perennail wildflower frequently used in traditional Chinese medicine (TCM). PV extracts can have multiple biological activities, including anti-inflammatory, anti-bacterial, and anti-cancer effects (1). PV is rich in phenolic acids, flavonoids, coumarins, triterpenes, volatile oil, and polysaccharides (2, 3). Furthermore, PV extracts have been reported to have chemopreventive potential in non-small cell lung cancer (4). The biological functions of PV are related to its immunomodulatory effects (5), including increased activity of natural killer (NK) cells and T lymphocytes, enhanced lymphopoiesis, and altered production of TNF-α, IL-1β, and IL-6 (6, 7). Recently, several reports have demonstrated that PV extract is effective against multiple cancers, such as breast cancer (8), thyroid cancer (9), lung cancer (4), and liver cancer (10).

Yin-Yang theory originated from ancient Chinese philosophy and was incorporated into TCM. According to TCM, diseases result from an imbalance between Yin and Yang. Recently, cancer-associated genes and proteins were reported to regulate different cancers in a Yin-Yang manner (11), and this theory has been applied in the use of Chinese herbal medicine to treat NSCLC (12). The theoretical basis for lung tumorigenesis is the lack of fundamental substances—Qi and Yin—within the lungs. Qi and Yin are the vital energies that help maintain physical function, akin to the immune system in Western medicine. Qi deficiency can trigger stagnation of pathological byproducts, such as phlegm, damp, heat, and blood, ultimately leading to tumor lesion formation. Therefore, TCM strategies to prevent or treat lung cancer are designed to reinforce essence and eliminate pathological byproducts.

A new lung cancer prevention formula called PV-1 was developed by combining PV with three other Chinese herbs, *Polygonum bistorta, Sonchus brachyotus* and *Dictamnus Dacrycarpus* (1). *Polygonum bistorta* belongs to the family Polygonaceae, and the biological activities of *Polygonum bistorta* include anti-inflammatory, antibacterial, antiviral, antioxidant, and anticancer effects (13–15). *Sonchus brachyotus* is found to inhibit growth and proliferation of cancer cells, suggesting that *S. brachyotus* may have a role in cancer prevention (16). *Dictamnus dasycarpus* is an effective treatment for eczema and headaches in China (17). Furthermore, *Dictamnus dasycarpus* has pharmacological anti-inflammatory, antifungal, and anticancer properties (18, 19). Together, *Polygonum bistorta*, *Sonchus brachyotus* and *Dictamnus dasycarpu*s contribute to heat-clearing and detoxification in the lungs.

In PV-1, *Prunella vulgaris* is the primary herb known to enhance anti-cancer immune activity. *Sonchus brachyotus* and *Dictamnus dasycarpus* both serve to reinforce essence and eliminate pathological factors. In addition to enhancing immune activity, these herbs clear heat, as well as soften and resolve hard masses. *Polygonum bistorta* cools and accelerates blood circulation. By addressing the pathological causes of lung cancer development, this formula is likely to serve an important role in preventing and treating this disease.

The purpose of this study was to investigate PV-1’s chemopreventive effects and mechanism of action in induced lung and oral cancer mouse models. Benzo[a]pyrene or vinyl carbamate was used to induce lung cancer, while 4-nitroquinoline-1-oxide was used to induce oral cancer. The work presented here demonstrates the ability of PV-1 to prevent cancer in these well-established animal models. This study shows a selective inhibitory effect of PV-1 on immune suppressive granulocyte-like myeloid derived suppressor cells (g-MDSCs). PV-1 was also improved the anti-tumor activity of the programmed cell death protein (PD-1) blockade in both lung and melanoma syngraft models. Thus, PV-1 is a novel and highly effective cancer chemopreventive formula that can facilitate the immunoprevention of multiple types of cancers.

## Materials and methods

### Reagents and animals

The PV-1 was prepared by Longhua Hospital, Shanghai University Traditional Chinese Medicine (China). Herbal components were provided by Shanghai Traditional Chinese Medicine Technology Co. Ltd. (SPH Hua Yu Chinese Herbs Co. Ltd., Shanghai, China), the formula was prepared in a GMP facility (National Traditional Chinese Medicine Pharmaceutical Engineering Technology Research Center, Building 4, No. 200, Newton Road, Zhangjiang Hi-Tech Park, Pudong, Shanghai, China), allowing for standardized preparation of the herbal materials. The composition of PV-1 includes: *Polygonum bistorta*, *Prunella vulgaris*, *Sonchus brachyotus*, and *Dictamnus dasycarpus* 2:1:1:1. The main components of PV-1 were characterized by LC-MS. The resulting powder was stored at −80°C and freshly dissolved into water before each use. Vinyl carbamate (VC) was purchased from Toronto Research Chemicals (Canada). VC was dissolved in saline and freshly prepared immediately before each use. Benzo[a]pyrene [B(a)P] , 4NQO and tricaprylin were obtained from Sigma (St. Louis, MO). Female A/J, C57BL/6 and Rag 2^-/-^ mice were purchased from Jackson Laboratories (Bar Harbor, ME). Lewis lung carcinoma (LLC) cells were obtained from American Type Culture Collection (ATCC). B16-BL6 (B16) melanoma cells were obtained from Dr. Li Wang (Lerner Research Institute) (20). Both LLC and B16 cells were cultured in RPMI-1640 (Thermofisher), which was supplemented with 10% fetal bovine serum and 1% penicillin/streptomycin. The animal studies were approved by the Houston Methodist Research Institute Institutional Animal Care and Use Committee (approval number: IS00006363).

### Toxicity studies of PV-1

Eight-week-old female A/J mice were used in the PV-1 toxicity study. Mice were treated with different doses of PV-1 by oral gavage, the duration was five times per week for eight weeks. Body weights were measured bi-weekly. One hour after the last treatment, serum was collected to test liver function (alanine transaminase (ALT) and aspartate aminotransferase (AST)) and kidney function (blood urea nitrogen (BUN)) at Marshfield Labs (Marshfield, WI) (21). In the meantime, plasma was collected in EDTA-treated tubes for the quantitative analysis of PV-1. The samples were analyzed with UPLC-MS/MS. Baohuoside I was used as an internal standard.

### Chemopreventive study of PV-1 in lung cancer mouse models

Animal studies with two different carcinogens were carried out. For the first experiment, A/J mice were given a single intraperitoneal injection of VC (16 mg/kg body weight) in saline. One week after the VC injection, mice were randomly divided into two groups, the vehicle control group and the PV-1 treatment group (n=12 per group). PV-1 (3000 mg/kg body weight) was given by oral gavage (0.3 ml once daily), five times per week, for 20 weeks; control mice were given the same volume of PBS. Body weights were recorded every week. Mice were analyzed for the number and/or size of tumors and imaged by MRI before being euthanized. A 9.4 T MRI (Bruker) with a custom birdcage style Quadrature coil (Doty Scientific) was used to monitor the tumors as described previously (22). Five lungs and spleens from each group were collected for flow cytometry analysis.

In the second experiment, A/J mice were given intraperitoneal injection of 100 mg/kg body weight B(a)P. B(a)P was dissolved in tricaprylin. Mice were randomly divided into two groups, the vehicle control group and PV-1-treated group (n=10 per group). Two weeks after B(a)P injection, mice were treated for 18 weeks as described above. Body weights were recorded every week. Tumor numbers and sizes were assessed at the experimental endpoint.

For both experiments, lungs from each mouse were fixed in zinc formalin for 24 hours and then stored in 70% ethanol. The lungs were evaluated with a dissecting microscope as previously described (23). The following formula: V= (4/3) πr^3^ was used to calculate the tumor volume, and the tumor load was determined by averaging the total tumor volumes of individual mouse in each group.

### Chemopreventive study of PV-1 in oral carcinogenesis

The 4NQO-induced mouse oral cancer model has been commonly used to assess the effect of natural compounds on oral carcinogenesis (24). Six-week-old C57BL/6 J mice were randomized into two groups: vehicle control group and PV-1-treated group. To drinking water, 4NQO (50 μg/mL) was provided to mice for 16 consecutive weeks. Mice were treated with PV-1 or vehicle one week after 4NQO treatment and continued for 20 weeks. Mice were treated five times per week. The body weights were measured weekly. Twenty weeks after the start of PV-1 treatment, mice were euthanized, and tongues were collected to measure oral tumor size.

### RNA processing and RNA-seq assays

The tumors out of three pairs of lungs from each group (control and PV-1-treated) of B(a)P-treated mice were harvested and pooled by individual mouse for RNA-seq analysis. The Qiagen (Valencia, CA) RNeasy^®^ Mini Kit was used to extract total RNA from the lung tissues. Agilent 2100 Bioanalyzer (Santa Clara, CA) was used for measuring the quality of the RNA samples. The HiSeq 2500 sequencing platform (Illumina, San Diego, CA) was used for whole transcriptome analysis of RNA-seq library samples, and the quality of the RNA-seq reads was analyzed using the FastQC program (http://www.bioinformatics.babraham.ac.uk/projects/fastqc/). The coverage ranged from 15 million to 32 million reads per sample. The quality scores of >95.3% of all the bases of each sample were at least 30, and averaged about 40, greatly exceeding the gold standard threshold of 20.

Raw RNA-seq reads were aligned to the mm9 mouse genome (UCSC version, July 2007) using Bowtie-TopHat (version 2.0.4) (25, 26). HTseq was used to obtain read counts (27) and the R package *RUVSeq* was used to adjust for batch effects (28). Data normalization and differential expression analysis were conducted using *edgeR* and *limma* (29, 30).

To determine marker genes for different immune cell populations, the tumor genes that were differentially expressed between the PV-1-treated and vehicle control mice were analyzed using gene set variation analysis (GSVA) (31). The R package *heatmap3* (https://cran.r-project.org/web/packages/heatmap3/) was used to generate gene expression heatmaps. Bar plots were generated using GraphPad Prism 9.0 software (https://www.graphpad.com/company/).

### Flow cytometry

Mouse lungs tumors were harvested and pooled from each mouse, processed into a single-cell suspension using a tumor dissociation kit (Miltenyi Biotec, CA). Spleens were collected and processed to single-cell suspensions. Cells were first stained with fixable live/dead dye in PBS, and after a PBS wash, they were stained with surface T cell-staining buffer containing fluorochrome-conjugated anti-CD45, anti-CD4, anti-CD8a, anti-CD44, and/or anti-CD62L antibodies; or with myeloid-staining buffer containing anti-CD11c, anti-CD11b, anti-Ly6G, anti-Ly6c, anti-F4/80, NKp46, and/or anti-MHCII antibodies.

To measure intracellular cytokines, after T cell surface staining, cells were washed, fixed, permeabilized, and then stained with intracellular cytokine staining buffer containing anti-granzyme B, anti-IFN-γ, and/or anti-TNF-α antibody. Cells were evaluated by an LSR-II flow cytometer (BD). FlowJo software was used for the analysis of the data (32).

### Combination of PV-1 with anti-PD-1 antibody in syngraft animal melanoma or lung models

To generate syngrafts in C57BL/6 mice, 3 × 10^4^ B16 melanoma cells or 1×10^5^ LLC cells (in 0.1 ml of PBS) were inoculated subcutaneously into the right flanks of mice. Animals were randomly assigned to one of four groups: 1) vehicle control; 2) PV-1 (3000 mg/kg/day); 3) 200 µg/mouse anti-PD-1 antibody (Biolegend, San Diego, CA) given by the intraperitoneal injection of every other day; or 4) combined PV-1 and anti-PD-1 antibody. For the tumor syngrafts in B16 Rag2^-/-^ mice, 6-week animals were given: 1) vehicle control; 2) PV-1 (3000 mg/kg/day); 3) anti-PD-1 antibody. Mice in LLC model were treated 16 days, and mice in B16 model were treated 3 weeks. Tumor sizes were measured with a digital caliper. The following formula (D × d2)/2, was used for tumor size, in which D represents the large diameter and d represents the small diameter of the tumor.

### Statistical analysis

GraphPad Prism 9.0 (GraphPad Software) was used for general statistical analyses between treatments. Student’s t-test was applied for pairwise comparisons. For assessing multiple comparisons, ANOVA was used with Tukey’s *post-hoc* test. P-values < 0.05 were considered significant. All data were presented as means ± standard error of means (SEM). Sample sizes (n) and replicates were mentioned on each figure legend. For all figures, *p < 0.05,**p <0.01, and ***p < 0.001 were considered statistically significant.

## Results

### Toxicity study of PV-1

**Figure 1:**
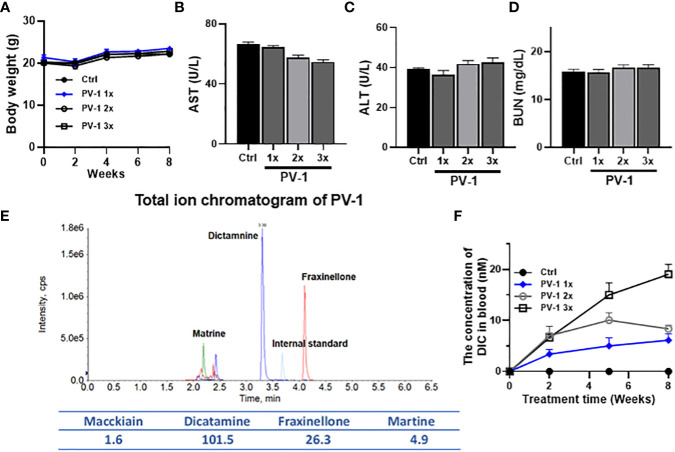
Toxicity study of PV-1. An 8-week toxicity study for PV-1 was conducted in A/J mice. Different doses of PV-1 were given *via* oral gavage 5 days/week **(A)** Body weights of treated and control mice. **(B-D)** Blood levels of liver function indicators (ALT, B; AST, C) and kidney function indicator (BUN, D) after 8 weeks of treatment with PV-1. The 1×, 2×, and 3× doses are relative to the treatment dose used in the efficacy study (1× = 3000 mg/kg). **(E)** Total ion chromatogram of PV-1. **(F)** The plasma concentrations of dictamine (DIC), a major component of PV-1, in mice from different dose treatment groups (n=5 per group).

To monitor the toxicity of PV-1, an eight-week study was conducted in A/J mice given either 1× (3000 mg/kg.bw), 2× (6000 mg/kg.bw), or 3× (9000 mg/kg.bw) five times per week of PV-1. Body weights were measured biweekly. Compared with the control group, treatment with different doses of PV-1 did not cause changes in body weight (**Figure 1A**). The general appearance of the animals (skin, eyes, breathing, and hair loss) and clinical signs for potential toxicities such as bleeding or diarrhea were also evaluated. No differences were observed between the PV-1-treated and control mice. Serum liver enzymes (ALT, AST) and BUN were also measured, and PV-1 did not change any of these liver or kidney function indicators (**Figures 1B-D**). These results suggest that PV-1 is safe and suitable for future clinical studies.

### Quantitative analysis of PV-1 exposure by content of marker compound dictamine

UPLC-MS/MS was used to analyze the components of PV-1 based on previous publications (33). The key components of PV-1 are identified as dictamine, maackiain, and matrine. Dictamine was selected to be used as a marker compound for PV-1 due to its bioactivity against cancer cells and its reasonable abundance in PV-1 (**Figure 1E**). Mice were treated with different doses of PV-1 for 8 weeks. One hour after the final treatment, dictamine plasma concentrations were assessed. Dictamine levels exhibited time- and dose-dependent increases in blood samples (**Figure 1F**).

### Inhibitory effect of PV-1 in lung tumor models

**Figure 2:**
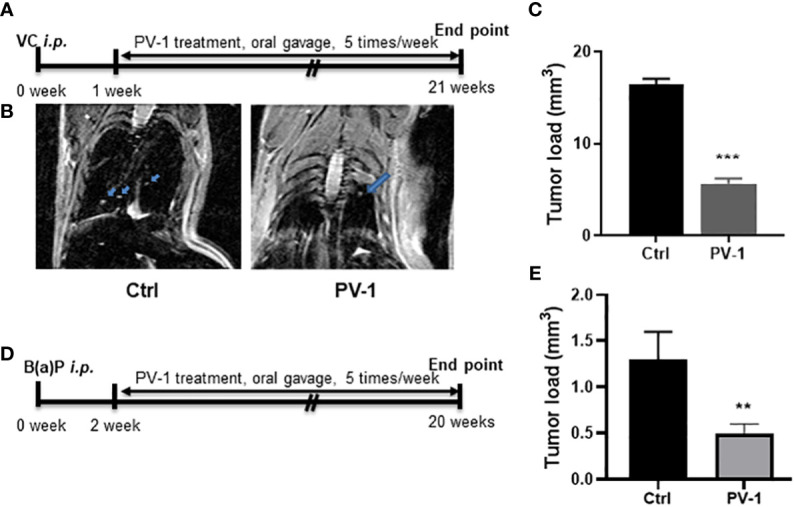
Anti-tumor efficacy of PV-1 in VC- and B(a)P-induced lung tumorigenesis. **(A, D)** Experimental design of the efficacy studies in VC **(A)** or B(a)P **(D)** tumor-induced mice. **(B)** Representative MRI images of VC-induced mice scanned 20 weeks after VC injection. Arrows indicate the tumors. **(C, E)** Effects of PV-1 on VC- induced **(C)** or B(a)P-induced **(E)** tumor loads at the time of euthanasia. *p < 0.05, **p < 0.01, ***p < 0.001 (n=12 per group in VC model, n=10 per group in B(a)P model).

There have been no prior publications demonstrating the impact of PV-1 on the development of lung tumors. Here, the chemopreventive effect of oral gavage PV-1 in VC- or B(a)P-induced lung adenocarcinoma in female A/J mice was investigated according to the experimental designs shown in **Figures 2A, D**. In both lung cancer models, tumor incidence was 100% for all treatment groups. There were no obvious abnormal manifestations such as hair loss, icteric sclera, or changes in body weight (data not shown). The absence of any clinical toxicity is consistent with the data in **Figure 1**.

In the VC-induced lung adenocarcinoma model, MRI imaging was used to evaluate tumor size. Observable differences were identified between control and PV-1-treated mice **(** **Figure 2B** **)**. At the experimental endpoint, tumor loads in the VC-induced control group averaged 16.4 ± 0.3 mm^3^ in size, while tumor loads in the PV-1 groups were significantly reduced to 7.6 ± 0.5 mm^3^ and 5.6 ± 0.1 mm^3^, respectively (**Figure 2C**). In the B(a)P model, tumor load in the control group was 1.3 ± 0.3 mm^3^, while mice treated with PV-1 showed a significantly decreased tumor load of 0.5 ± 0.1 mm^3^ (62.0% inhibition) compared with the controls (**Figure 2E**).

### PV-1 modulates immune cell populations in the tumor microenvironment

**Figure 3:**
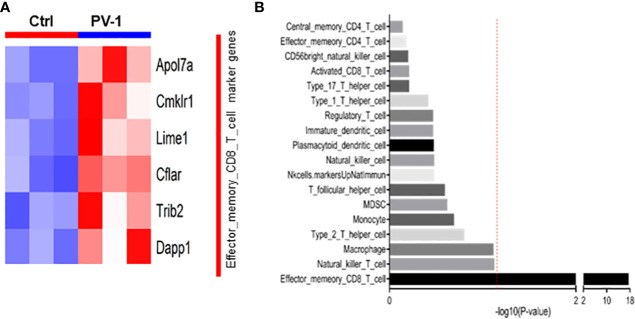
RNA-seq analysis indicates increased immune cell gene expression after treatment with PV-1. **(A)** Heatmap of the eight differentially expressed marker genes for the effector-memory CD8+ T cell population, of which six were upregulated by PV-1 treatment. **(B)** GSVA indicated that the effector-memory CD8+ T cell population was significantly increased in mouse tumors after PV-1 treatment, when compared with vehicle controls. The red line indicates the cutoff P value of 0.05 versus control mice (n=3).

Mouse tumors were collected from control and PV-1-treated mice in the B(a)P model for RNA-seq and assessed for changes that would indicate effects on immune cell populations. PV-1 caused an upregulation of six marker genes known to be expressed in effector-memory CD8+ T cells (**Figure 3A**). GSVA levels indicated that the effector-memory CD8+ T cell population was significantly more activated in tumors of mice treated with PV-1 than in vehicle controls (**Figure 3B**). These results suggest that the anti-tumor efficacy of PV-1 could involve the modulation of immune cells in the tumor microenvironment, specifically the activation of effector T cells.

**Figure 4:**
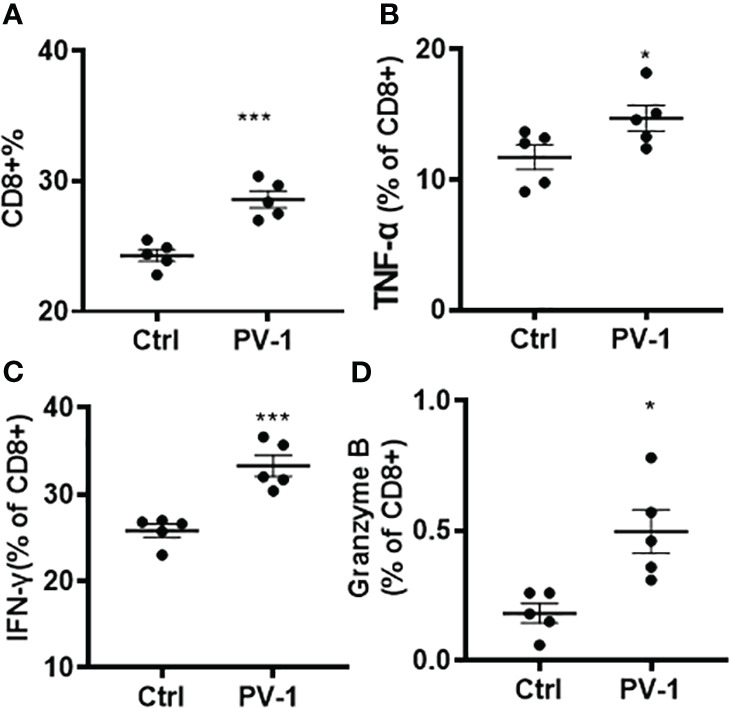
Analysis of tumor-infiltrating CD8+ T cells. **(A)** Percentages of CD8+ tumor-infiltrating lymphocytes. **(B–D)**. Granzyme B, TNF-α and IFN-γ expression in CD8+ cells. The Mann-Whitney two-tailed unpaired t-test was used to determine statistical significance, *p < 0.05, **p < 0.01, ***p < 0.001 (n=5).

**Figure 5:**
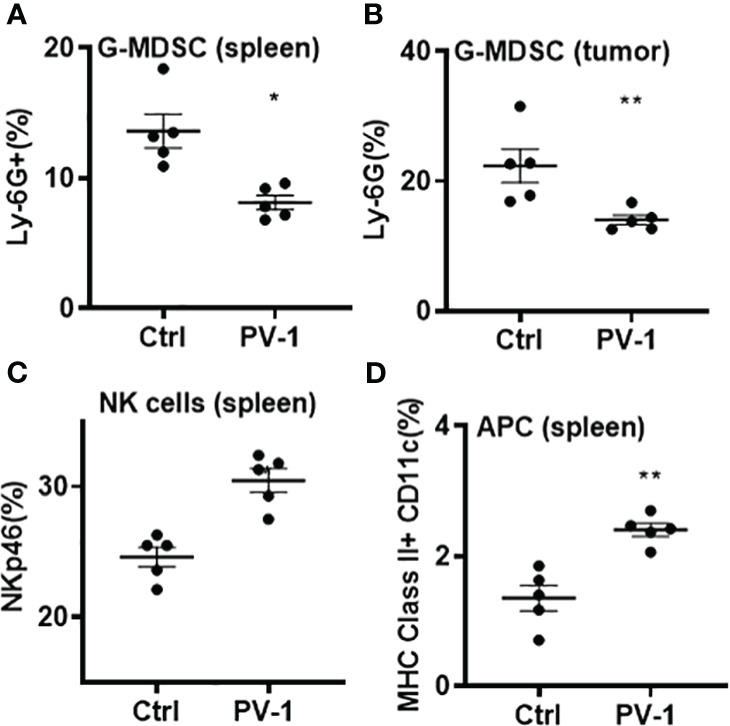
Analysis of g-MDSCs, NK cells and APC from control and PV-1-treated mice by flow cytometry. **(A**, **B)** Percentages of g-MDSCs in spleens and lung tumors. **(C, D)** Percentages of NK cells and antigen-presenting cells in spleens. The Mann-Whitney two-tailed unpaired t-test was used to determine statistical significance, *p < 0.05, **p < 0.01 (n=5 per group).

To further explore whether PV-1 modulates immune function, flow cytometry was conducted on tumors and splenocytes isolated from VC-induced mice using various immune cell markers. In the lung tumors from VC-induced mice, the percentage of CD8+ tumor-infiltrating lymphocytes (TILs) was significantly increased upon PV-1 treatment (**Figure 4A**), and the production of granzyme B, TNF-α and IFN-γ by CD8+ cells were all significantly increased in these mice (**Figures 4B-D**). The g-MDSCs (CD11b+Ly6G+) were significantly decreased in the spleens and tumors of PV-1-treated mice compared with controls (**Figures 5A, B**). In addition, significant increases in the percentages of natural killer (NK) cells and antigen-presenting cells (APC) were found in mouse spleens after treatment with PV-1 (**Figures 5C, D**). Because MDSCs can markedly suppress the T cell response, PV-1-mediated suppression of MDSCs could enhance the function of T cells that target tumor cells. These results suggest that the cytotoxic function of these CD8+ cells is increased by PV-1, which could facilitate their migration to tumor sites and increase their potential to kill tumor cells.

### PV-1 improves the anti-tumor efficacy of anti-PD-1 antibody in a syngraft model

**Figure 6:**
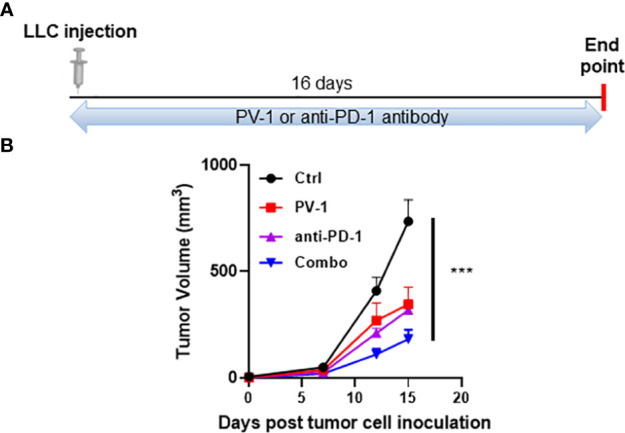
PV-1 improves the anti-tumor efficacy of anti-PD-1 antibody in the LLC model. **(A)** Experimental design: 1× 10^5^ LLC cells were inoculated subcutaneously into the right flanks of experimental and control mice. Animals were randomly assigned to four groups that were given vehicle control, PV-1 (3000 mg/kg/day), anti-PD-1 antibody, or a combination of PV-1 and anti-PD-1 antibody. **(B)** Tumor growth curves for each treatment group of mice. ***p < 0.001 (n=5).

Clinical studies have demonstrated that PD-1/PD-L1 antibodies/inhibitors induce anti-tumor efficacy in non-small cell lung cancer (34, 35). Therefore, an LLC syngraft mouse model was used to determine whether PV-1 could enhance the efficacy of the anti-PD-1 checkpoint blockade. Subcutaneous injection of LLC tumor cells was done on the flanks of C57BL/6 mice, and mice were treated with PV-1, anti-PD-1 antibody, or both in combination, five times/week for 16 days (**Figure 6A**). Treatment with PV-1 alone inhibited tumor growth by 53.8%, while treatment with anti-PD1 antibody alone inhibited tumor growth by 56.7%, when compared with the control (**Figure 6B**). Notably, a significantly greater anti-tumor effect was observed with the combination of PV-1 and anti-PD1 antibody (75.4% inhibition vs. control).

**Figure 7:**
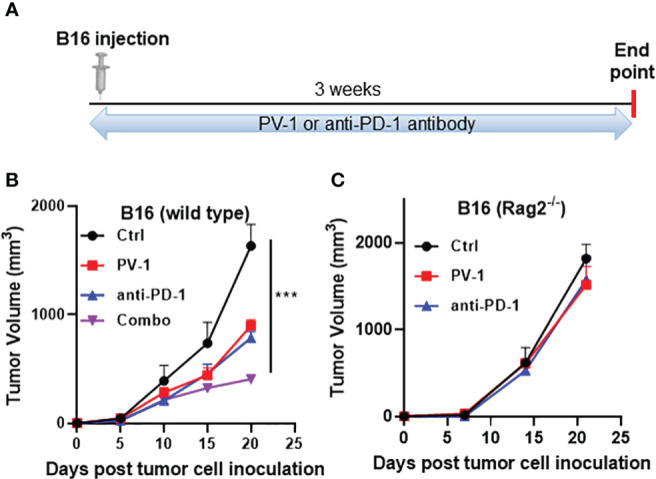
Adaptive immunity is necessary for the anti-tumor effects of PV-1 in a melanoma syngraft model. **(A)** 3× 10^4^ B16 melanoma cells were inoculated subcutaneously into the right flanks of experimental and control mice. Animals were randomly assigned to four groups: vehicle control, PV-1 (3000 mg/kg/day), anti-PD-1 antibody, or a combination of PV-1 and anti-PD-1 antibody. **(B)** Tumor growth curves for each group in C57BL/6 mice. **(C)** Tumor growth curves of each treatment group in Rag2^-/-^ mice. ***p < 0.001 (n=5).

To confirm that the anticancer effects of PV-1 are mediated through adaptive immune mechanisms, the effects of PV-1 and anti-PD-1 antibody were measured in B16-inoculated Rag2^-/-^ mice (**Figure 7A**), which do not contain mature B or T lymphocytes (36). Like the LLC model, inhibition of B16 tumor volumes in wild-type mice by PV-1 or anti-PD1 antibody alone were 45.0% and 52.1%, respectively (**Figure 7B**). The combination of PV-1 and anti-PD1 antibody induced better anti-tumor efficacy than either agent alone (75.0% inhibition) (**Figure 7B** **)**. In Rag2^-/-^ mice, neither PV-1 nor anti-PD-1 antibody treatment induced significant tumor inhibition (**Figure 7C**). These results indicate that the anti-tumor effects of PV-1 are heavily dependent on adaptive immunity.

### Inhibitory effect of PV-1 in the oral carcinogenesis model

**Figure 8:**
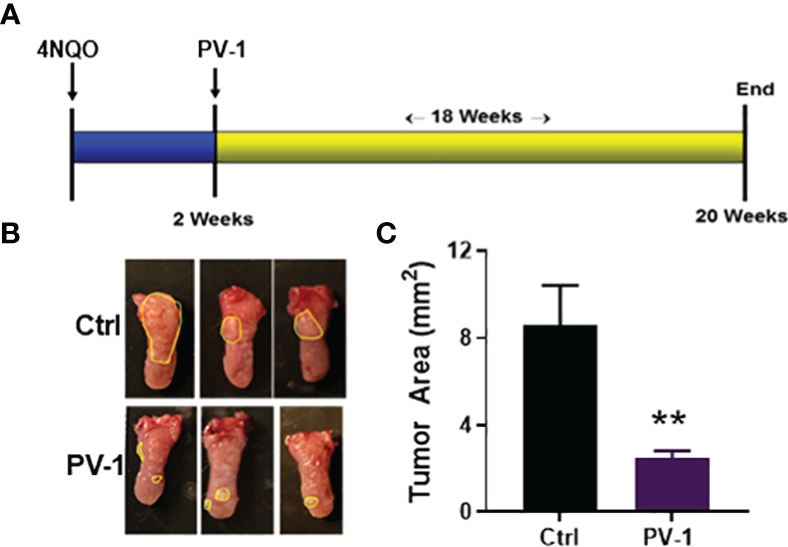
Inhibitory effect of PV-1 on 4NQO-induced oral cancer. **(A)** Experimental design. **(B)** Representative images of 4NQO-induced lesions. **(C)** Tumor area per mouse PV-1-treated and control mice. **p < 0.01 (n=12).

To test the impact of PV-1 on 4NQO-induced oral carcinogenesis, C57BL/6 mice were treated as illustrated in **Figure 8A**. Compared with the control group, mice treated with PV-1 showed no difference in body weight (data not shown), and consumption of water was similar between the control and treated groups. Tongues of 4NQO-treated mice showed white spots or exogenous papillary lumps (**Figure 8B**). PV-1 treatment induced significant inhibition of tumor lesions when compared with controls; the mean tumor area decreased from 8.1 mm^2^ in the 4NQO control group to 3.8 mm^2^ (53.1% inhibition) in the PV-1 group (**Figure 8C**).

## Discussion

Previous studies have shown that *Prunella vulgaris* (PV) has significant immune-stimulatory and anti-inflammatory effects in mouse models (6, 7). Here, the effects of PV-1, a novel *Prunella vulgaris*-containing formula that also includes three other botanicals, *Polygonum bistorta, Sonchus brachyotus* and *Dictamnus dasycarpus*, was examined in different mouse cancer models. The results indicate that PV-1 is a safe and highly effective chemopreventive against lung and oral cancers. PV-1 can also provide a synergistic anti-tumor effect when combined with anti-PD-1 immunotherapy. PV-1 significantly suppressed lung tumor formation in two different mouse adenocarcinoma models. These results suggest that the anti-tumor effects originate from the ability of PV-1 to increase tumor-infiltrating CD8+ cytotoxic T cell numbers. PV-1 was also able to enhance T cell function, as demonstrated by increased IFN-γ, granzyme B, and TNF-α expression. Meanwhile, the frequency of immunosuppressive g-MDSCs was significantly decreased in PV-1-treated tumors, suggesting that PV-1 might enhance T cell function by reducing the numbers of g-MDSCs in the tumor microenvironment. Finally, PV-1 did not cause detectable toxicity (**Figure 1**) and induced strong efficacy against 4NQO-induced oral tumors (**Figure 8**). These findings indicate that PV-1 may be a good candidate for testing in chemoprevention clinical trials.

The *in vivo* anti-tumor efficacy of PV-1 may be mediated through its ability to enhance the activity of immune cell populations, including effector-memory CD8+ T cells, as revealed by RNA-seq analysis. Using flow cytometry, PV-1 was found to induce significant decreases in the frequencies of both splenic and tumor g-MDSCs, resulting in increased expression of granzyme B, TNF-α and IFN-γ by CD8+ cells (**Figure 5**). Using immune checkpoint proteins like PD-1/PD-L1 has demonstrated considerable success in treating certain types of solid tumors (37). However, blocking PD-1/PD-L1 is not a universally successful cancer treatment, particularly for those cancers known to be resistant to immunotherapy. Thus, improving the response rates of tumors to immunotherapy is a major clinical goal. When combined in the mouse lung LLC model, PV-1 and anti-PD-1 together exhibited significantly better anti-tumor efficacy than either treatment alone. The results from studies in Rag2^-/-^deficient mice implicate the importance of adaptive immunity to the mechanism of action of PV-1, quite possibly *via* the T cell response. While T cells are known to be important to the anti-tumor effects of PD-1 blockade, a role for B cells cannot be ruled out.

Inhibiting g-MDSCs is also a promising approach to modulating tumor immunity for treating cancer (38). PV-1 treatment decreased the percentages of g-MDSCs in both tumors and spleens (**Figure 5**). Thus, additional experiments were used to investigate whether PV-1 combined with anti-PD-1 antibody could enhance the anti-tumor effects of anti-PD-1 antibody, at least partially through g-MDSC inhibition of T cells. Collectively, these studies suggest that PV-1 exerts its chemopreventive effect through the activation of effector-memory CD8+ T cells and inhibition of MDSCs.

In conclusion, this study demonstrates that PV-1 has pronounced anti-tumor efficacy, preventing lung and oral tumorigenesis with no obvious toxicity in multiple mouse models. Its anti-tumor effects may at least partially be due to its ability to increase CD8+ T cell infiltration into tumors and reduce suppressive g-MDSCs. This study also demonstrates that combination with anti-PD-1 antibody and PV-1 leads to enhanced preventive efficacy in murine tumor models. The results indicate that PV-1 could be a potential chemopreventive agent for lung and oral cancers, either alone or in combination with anti-PD-1 immunotherapy.

## Data availability statement

The data presented in the study are deposited in the NCBI BioProject database, accession number PRJNA998439.

## Ethics statement

The animal study was reviewed and approved by Houston Methodist Research Institute Institutional Animal Care and Use Committee, (approval number: IS00006363).

## Author contributions

QZ and JP were responsible for the overall experimental design with input by XC, KP, and DB. MY contributed to the overall concept, designed the formulation of PV-1, and supervise the execution of all experiments. The project was also supervised by BJ, MH, and YW. QZ and XC assessed anti-cancer efficacy in animal models. JP and KP conducted flow cytometry analysis, DB assessed PK analysis. DX conducted RNA-seq analysis. The following were largely responsible for writing, reviewing, and editing the manuscript: QZ, BJ, YW and MY. All authors contributed to the article and approved the submitted version.
